# *Homo floresiensis* Contextualized: A Geometric Morphometric Comparative Analysis of Fossil and Pathological Human Samples

**DOI:** 10.1371/journal.pone.0069119

**Published:** 2013-07-10

**Authors:** Karen L. Baab, Kieran P. McNulty, Katerina Harvati

**Affiliations:** 1 Department of Anthropology and Interdepartmental Doctoral Program in Anthropological Sciences, Stony Brook University, Stony Brook, New York, United States of America; 2 Evolutionary Anthropology Laboratory and Department of Anthropology, University of Minnesota, Minneapolis, Minnesota, United States of America; 3 Department of Early Prehistory and Quaternary Ecology, Senckenberg Center for Human Evolution and Paleoecology, Eberhard Karls University of Tübingen, Tübingen, Germany; Illinois State University, United States of America

## Abstract

The origin of hominins found on the remote Indonesian island of Flores remains highly contentious. These specimens may represent a new hominin species, *Homo floresiensis*, descended from a local population of *Homo erectus* or from an earlier (pre-*H. erectus*) migration of a small-bodied and small-brained hominin out of Africa. Alternatively, some workers suggest that some or all of the specimens recovered from Liang Bua are pathological members of a small-bodied modern human population. Pathological conditions proposed to explain their documented anatomical features include microcephaly, myxoedematous endemic hypothyroidism (“cretinism”) and Laron syndrome (primary growth hormone insensitivity). This study evaluates evolutionary and pathological hypotheses through comparative analysis of cranial morphology. Geometric morphometric analyses of landmark data show that the sole Flores cranium (LB1) is clearly distinct from healthy modern humans and from those exhibiting hypothyroidism and Laron syndrome. Modern human microcephalic specimens converge, to some extent, on crania of extinct species of *Homo*. However in the features that distinguish these two groups, LB1 consistently groups with fossil hominins and is most similar to *H. erectus*. Our study provides further support for recognizing the Flores hominins as a distinct species, *H. floresiensis*, whose affinities lie with archaic *Homo*.

## Introduction

The discovery of a small-bodied, small-brained hominin in Liang Bua cave on the remote Indonesian island of Flores [1] sparked a highly contentious and still unresolved debate within the paleoanthropological community [2]–[14]. Brown and colleagues initially attributed the remains to a new hominin species, *Homo floresiensis* [1]. On the basis of the primitive craniodental and postcranial morphology, including an estimated cranial capacity of only 380–430 cm^3^ [1], [4], [15], [16], they suggested that this species was the descendent of either *Homo erectus* (via island dwarfing) or of an as yet undocumented small-bodied and small-brained hominin from the Sunda Shelf [1]. Other workers came to the very different conclusion that the remains of one or more individuals from Liang Bua cave represent pathological modern humans. Thus, the most complete specimen, LB1, has been labeled with a variety of potential pathological conditions, including microcephaly [6], [10], [12], [16], [17], congenital hypothyroidism [18], [19], and Laron syndrome [20]. The presence of a late-surviving, small-brained and small-bodied archaic hominin on Flores would have far-reaching implications for our understanding of human evolution, pointing to a previously unidentified, ancient lineage of hominins that persisted long after the appearance of modern humans in Southeast Asia. Therefore, a thorough evaluation of alternative hypotheses about LB1's status is imperative.

The initial proposal that *H. floresiensis* descended from *H. erectus* rested on craniofacial similarities – such as a low cranial vault, thick cranial bones, a frontal keel, mastoid fissure and a fissure between the tympanic plate and entoglenoid pyramid [1] – as well as biogeographical considerations: *Homo erectus* is the only hominin known from island Southeast Asia prior to the arrival of modern *Homo sapiens* around 60–45 ka [21], [22]. At the same time, postcranial and dentognathic characters of the Liang Bua fossils imply either a pre-*H. erectus* ancestry or a series of evolutionary reversals from a *H. erectus* predecessor [23], [24]. In particular, the shape of the mandibular symphysis, limb proportions, interlimb strength ratio and possibly the P_3_ crown/root morphology, flaring iliac blades, and carpal/tarsal morphology are less derived in Liang Bua specimens than in available *H. erectus* specimens [11], [23], [25]–[27]. Although LB1’s endocranial volume is smaller than any known *H. erectus* or *Homo habilis* specimen, its endocast morphology aligns it with *Homo* rather than *Australopithecus* [4,5, but see 28].

Quantitative analyses have likewise confirmed that the LB1 skull is more similar to those of archaic *Homo* than *Australopithecus* [2], [3], [29]. There is no agreement, however, as to which species it most closely resembles. Several analyses likened its cranial shape to geochronologically older *H. erectus* fossils from Africa and Georgia, and, to a lesser extent, *H. habilis* [2], [30]. Another found that LB1 was most similar to early Javanese *H. erectus* [31]. A cladistic analysis grouped *H. floresiensis* with species of early *Homo*, and indicated a likely divergence prior to the appearance of *Homo ergaster/H. erectus* [5], [32].

In contrast, critics of the “new species” hypothesis have pointed out that the degree of brain size reduction implied by the dwarfing scenario is inconsistent with empirical observations of island dwarfing in other mammals [10, but see 15,33], and that the shape of the brain in LB1 resembles that of microcephalic modern *H. sapiens* [10], [28]. Jacob et al. [16] also argued that many of the traits proposed to distinguish the Liang Bua remains from *H. sapiens* can be found in contemporary Australo-Melanesian populations, and that it is unlikely that a small-brained hominin was capable of producing the lithic artifacts found in the same stratigraphic layers of Liang Bua cave [10, but see 5]). Moreover, some of the apparently primitive postcranial features identified in the Liang Bua specimens have been attributed to specific pathological conditions [e.g., humeral head torsion; 19,20,34], and craniofacial and postcranial asymmetries have been cited as indicative of a serious developmental disorder [16,17, but see 35].

These workers interpret the LB1 skeleton as a pathological modern human, possibly deriving from a small-bodied insular population. Early arguments in this vein labeled LB1 with microcephaly [6], [10], [12], [16], a clinical sign where abnormal brain growth results in a small cranial vault [one to three standard deviations below the appropriate age and sex averages; 36]. Although microcephaly can occur in isolation, it is sometimes associated with short stature [36]–[38], an observation with particular relevance to LB1 with an estimated stature of only ∼106 cm [1]. In addition to a small neurocranium, many (but not all) microcephalic skulls exhibit a sloping forehead (frontal bone) [39]–[41], which is also seen in LB1.

A second pathology proposed to explain LB1’s anatomy is ME hypothyroidism [18], [19], in which a lack of dietary iodine, pre- and post-natally, leads to improper functioning of the thyroid gland [42]. Individuals with ME hypothyroidism exhibit reduced stature (thyroid hormone deficiency adversely affects endochondral bone development) and sometimes reduced brain size. Advocates of this hypothesis have identified additional resemblances between LB1 and “cretins,” including a large pituitary fossa, open bregmatic fontanelle, absent frontal sinuses, retention of deciduous dentition in adults, delayed development of the clavicle and scapula, high humerofemoral index, and reduced humeral torsion [18,19, but see 43]. A final condition proposed for LB1 is Laron syndrome, an endocrine disorder characterized by high serum growth hormone levels but defective growth hormone receptors, which results in stunted growth due primarily to foreshortened legs [20]. Clinical manifestations of Laron syndrome are similar to other endocrine disorders that lead to short stature, including congenital growth hormone deficiency and IGF-1 gene deletion [44]. Characters cited in support of this diagnosis include a small mandible lacking a mental protuberance, small skull, thick humeral shafts and low humeral torsion angles [20].

Detailed responses to the Laron syndrome and cretinism arguments have been published, contesting the accuracy of the anatomical observations for LB1 or the interpretation of these features as pathological. Falk et al. [45] presented evidence that directly contradicted many of the standard clinical criteria used to diagnose Laron syndrome in the Flores skeletal remains (e.g., protruding forehead, small feet/hands, and an absent frontal sinus). These authors also argued that features used by Hershkovitz et al. [20] to diagnose LB1 with Laron syndrome are not part of the standard clinical manifestations of this disorder (e.g., normal cranial bone thickness, size and shape of the clavicle).

Likewise, metric and nonmetric comparisons of the Flores hominins to patients with ME hypothyroidism [43] failed to corroborate the claims of Obendorf et al. [18] that LB1 and LB6 suffered from “cretinism.” Of relevance to the current study, Brown [43] found no evidence for ME hypothyroidism in the LB1 skull, citing, for example, a markedly reduced endocranial volume, a normal-sized sella turcica, and well-developed paranasal sinuses in LB1 and in contrast to many ME hypothyroidism patients. However, more recent work by Oxnard and colleagues [19], [34] has taken issue with Brown [43], and presented new evidence, primarily from the postcranial skeleton, supporting ME hypothyroidism. While the findings in this recent study have yet to be fully evaluated by the scientific community, Orr et al. [27], using new fossil specimens and a large comparative sample, have shown that the wrist morphology of the Flores hominins is likely plesiomorphic rather than pathological [contra 17, 18].

The hypothesis that LB1 exhibits evidence of microcephaly is more challenging to counter, as microcephaly is associated with many different disorders. For this reason, Falk’s [4] original comparative analysis of LB1 was criticized because it included only one microcephalic individual, and therefore did not represent the true heterogeneity of microcephalic endocranial morphology [12], [46]. Subsequently, Falk et al. [47] were able to increase their microcephalic sample to nine individuals, and argued that a few key aspects of brain shape consistently differentiated LB1 from all microcephalic individuals. These results were questioned by Vannucci et al. [28] on the basis of craniometric dimensions taken from a large sample of both microcephalic and normocephalic individuals. However, this sample comprised many infants and children, complicating interpretations of the results. Two analyses of ectocranial dimensions have rejected the hypothesis of microcephaly for LB1, but both were based on very small sample sizes for the microcephalics [2], [30].

Hence, the status of the LB1 cranium is still unresolved, and the persistence of these competing hypotheses is due in part to the fact that most analyses have assessed its craniofacial affinities in either exclusively phylogenetic or exclusively pathological contexts [3], [16], [18], [20], [29]–[31], [48]. Those few studies that combined phylogenetic and pathological comparisons suffered from very small sample sizes and were not able to include the full range of proposed pathological conditions [2], [30]. Here, we provide a new morphometric evaluation of both phylogenetic and pathological hypotheses for LB1 by comparing its neurocranial shape to phylogenetically relevant hominins and to a large sample of pathological individuals representing each of the implicated conditions.

Analyzing the cranium is particularly appropriate given that craniofacial morphology has figured prominently in explanations of the Liang Bua hominins and because cranial morphology, particularly neuro- and basi-cranial shape, have been shown to be phylogenetically informative in previous studies of Pleistocene hominins [49]. Statistical analyses of cranial morphometric data also provide an alternate means of assessing pathologies – one that circumvents debates over more subjective definitions, identifications, and interpretations of these conditions in the Liang Bua material [18]–[20], [43], [45]. Furthermore, the larger samples used here of humans with microcephaly and ME hypothyroidism better capture the heterogeneity in these disorders, and thereby provide a more robust assessment of the likelihood that LB1 suffered from one of them.

## Materials

Data on LB1 were collected from a stereolith model (based on computed tomography scans) created by P. Brown and ARKANAS that has been used in previous studies of LB1 [3], [28]. The CT data that were the basis for this model were acquired in 2004 prior to the damage that occurred to the specimen after its discovery (although this damage appears to have only minimally impacted the linear dimensions of the LB1 cranium [31]). Stereolith models are routinely used for planning surgery and are therefore necessarily dimensionally accurate [50]–[52]. Linear dimensions measured from the stereolith model of the LB1 cranium are, on average, 0.5 mm greater than those taken on the original (range: −1 to +2 mm) [53]. These small differences are unlikely to influence the results of the analyses presented here. While concerns have been raised about the preservation conditions of LB1 upon its discovery [18], [34], analyses of cranial asymmetry suggest that its level of asymmetry is comparable to other wild-shot, non-pathological African ape crania and to well-studied – and presumably non-pathological – fossil hominin crania [3], [35]. As discussed by Kaifu et al. [8], [54], the deformation that *is* present in the LB1 cranium can probably be explained by positional deformational plagiocephaly, a condition that results from plastic deformation of the skull during infancy, but without serious health effects.

Our non-pathological modern human comparative data included geographically widespread samples of recent populations from Africa (Afalou, Taforalt, Teita, Khoe-San), the Middle East (Lachish), Europe (Greifenberg), Asia (Chinese, Mongolian, Andamanese), Australia (aboriginal Australian) and North America (Grand Gulch, Point Hope Inuit) (n = 194), as well as the late Pleistocene modern *H. sapiens* specimens Skhul 5 and Cro Magnon I (Table 1). Both the Khoe-San and Andaman Islanders are small-bodied populations. Fossil hominin data were collected from the following species: *H. habilis*, *H. erectus sensu lato*, *H. heidelbergensis s.l.* and *H. neanderthalensis*. Data were collected from original fossil specimens when possible, and casts when the originals were not available (specifically the Dmanisi and Zhoukoudian samples, Sangiran 17, Dali, Sima de los Huesos 5, La Chapelle aux Saints, and La Ferrassie 1) (n = 20) (Table 1). Permission/permits to study the hominin fossils and casts as well as the healthy modern human comparative material (which included archaeological material) were granted by the following institutions and authorities: National Museums of Ethiopia, National Museums of Kenya, the Kenya Ministry of Education, Science and Technology, University of Cape Town, Iziko Museums of Cape Town (South African Museum), Gadja Mada University, Lembaga Ilmu Penelitian Indonesia (LIPI), Natural History Museum (London), University of Cambridge, Musée de L’Homme, Institut de Paleontologie Humaine, Peabody Museum of Archaeology and Ethnology, and the American Museum of Natural History. All necessary permits were obtained for the described study, which complied with all relevant regulations.

**Table 1:** Information about samples used in this study.

Sample	Sample size	Sources^1^	Data type	*A priori* assignment for between-group PCA
*Homo habilis*	1	NMK	Original	Not included
*Homo erectus*	13	AMNH, NMK, GMU, NME	Original/Casts	*Homo erectus*
*Homo heidelbergensis*	4	AMNH, NME, NHM	Original/Casts	mid-Pleistocene *Homo*
*Homo neanderthalensis*	2	MH	Casts	mid-Pleistocene *Homo*
*Homo sapiens*	227			
Upper Paleolithic	4	PM, MH	Original/Casts	Healthy *Homo sapiens*
Recent	223			
Healthy	192	AMNH, NHM, IPH, UCT, DC	Original	Healthy *Homo sapiens*
ME hypothyroidism^2^	10	NMB, MM	Original	ME hypothyroidism
?Sporadic hypothyoidism	4	NMNH, MH	Original	Not included
Laron syndrome	1	TAU	CT scans	Not included
?Growth hormone deficiency	1	NMNH	Original	Not included
Primary microcephaly	15	AMNH, PM, NMNH, MLU, UM, UV, WU, MH	Original/Casts/CT scans/Surface scans	Microcephaly
Secondary microcephaly	17	NMNH, MM, UM, UV, MH	Original/CT scans	Not included
Liang Bua hominins	1	INCA	Stereolith cast	Not assigned
TOTAL	248			

1, NMK: National Museum of Kenya; AMNH: American Museum of Natural History; GMU: Gadja Mada University; NME: National Museum of Ethiopia; NHM: Natural History Museum (London); MH: Musee de l'Homme; PM: Peabody Museum (Harvard University); IPH: Institut de Paleontologie Humaine; UCT: University of Cape Town; DC: Duckworth Collection (Cambridge University); NMB: Naturhistorisches Museum Basel; MM: Mutter Museum (Philadelphia); TAU: Tel Aviv University; MLU: Meckelsche Sammlungen, Martin-Luther Universität of Halle-Wittenberg, scanned at the Paleoanthropology High Resolution Tomography Laboratory, University of Tübingen; UM: University of Michigan; UV: University of Vienna; WU: Washington University; INCA: Indonesian National Center for Archaeology.

2, ME: myxoedematous endemic.

The modern human pathological samples included individuals exhibiting hypothyroidism, Laron syndrome, and microcephaly (primary and secondary). The sample of humans with ME hypothyroidism comprised individuals of Swiss descent as endemic cretinism has been documented in the Swiss Alps (n = 10). Eight of the ME hypothyroidism specimens are from the Galler Collection (NMB specimens 64, 65, 68, 84, 85, 85-a, 135, and 136), and two are from the MM (1006.006 and 1006.007) (See Table 1 for institution abbreviations). Three of the individuals from the Galler Collection were associated with autopsy records that diagnosed them as “cretins” or “dwarf cretins” [55] while the other five are associated with short stature and display other cranial and/or postcranial anomalies associated with this disorder [55]. Ortner and Hotz concluded that “it seems likely that these five cases were examples of endemic hypothyroidism” [55, p. 3]. Our analyses also included four specimens that are likely cases of sporadic hypothyroidism. One individual from the Galler Collection was diagnosed at autopsy as athyroid, and hence a case of sporadic hypothyroidism (NMB specimen 66). Another specimen from the Terry Collection (specimen number 1636), a collection of cadaver-derived skeletons from medical schools in Missouri, was diagnosed by Ortner [56] as having hypothyroidism. It is possible that this individual was a recent immigrant from a region with ME hypothyroidism, but we are unable to confirm this and hence included it in the sporadic hypothyroidism group. An additional cranium from New Mexico (NMNH specimen 271813) was identified by Ortner as a probable case of hypothyroidism [57], although he could not exclude the possibility of a rare chondrodysplasia. As ME hypothyroidism is not documented from New Mexico, this individual was included in the sporadic hypothryroidism group as well. Finally, an additional skull identified as a “cretin” in the Museé de l’Homme catalog (29518) had no accompanying locality information, and was thus included here with the sporadic hypothyroidism group. In both ME and sporadic hypothyroidism, insufficient thyroid hormone is produced, and there is some indication that the manifestations of both disorders in the skull are similar [58].

Laron syndrome was first described in 1966 [44] and is therefore not indicated in museum collections; CT scan data for a single individual with Laron syndrome were graciously provided by Z. Laron and L. Kornreich. A second cranium from a specimen tentatively identified as a “pituitary dwarf” at the NMNH (300R) was also included as the clinical manifestations of Laron syndrome are very similar to those seen in congenital growth hormone deficiency [44], [59], [60]; these are labeled separately in ordination plots.

Data were also acquired from 30 crania identified as microcephalic modern humans in catalog records at ten institutions in the United States and Europe, including CT scan data provided by D. Falk and K. Smith (see Table 1 and [4] for additional details). To distinguish between primary and secondary microcephaly in specimens with known endocranial volumes (EV), we used a threshold of 650 cm^3^ as a conservative upper bound for primary microcephaly [47]. A comparable threshold was also calculated for neurocranial size based on a regression of log(neurocranial centroid size) on log(EV) within this subset (*R^2^* = 0.90, *p*<0.0001). Specimens for which EV was unknown were thereby assigned to the primary microcephaly group if their neurocranial size yielded an EV estimate below 650 cm^3^. Based on known or estimated EVs, then, 14 crania were probable cases of primary microcephaly (AMNH: 99.7/2601 and Jakob Moegle cast; PM: 7200, 7387; MLU: 131, 140, 141; UM: 96-11-128A; WU cast; MH: 27422, 6288, 30212, 3486; UV: 5385), while the remaining 14 were provisionally assigned to the secondary microcephaly group (MM: 1006.052; UM: 660; UV 3795; MH: 5552, 5638, 24628, 25298, 29406, 29409, 29410, 29411, 29412, 29422). Hrdlička [61], [62] previously argued that one of the crania assigned here to the primary microcephaly group and two assigned to the secondary microcephaly group did not have microcephaly. Specifically, he suggested that a very small individual from Peru (EV of only ∼485 cm^3^; NMNH: 379510) was a “midget” (his description suggests that a “midget” was a proportional dwarf) [63]. In contrast, several other workers have favored diagnoses ranging from congenital idiocy to microcephaly [57], [62], [64], [65]. We included this cranium in our primary microcephaly sample due to its very small size and in the interests of maximizing variation in the microcephalic sample. Hrdlička argued that four additional crania from Peru with EVs ranging from 910 to 955 cm^3^ (two of which were labeled as microcephalics at the NMNH) fell at the extreme small end of the healthy human cranial size distribution in this population but are otherwise anatomically normal [61]. However, cranial capacities in this range are more than 3 standard deviations below the average EV for South American populations (*µ* = 1350 cm^3^, *σ* = 42) reported by Beals et al. [66], suggesting that the head circumferences would also be in the range of clinically identified microcephaly [38]. Therefore, these four crania were included in our study within the secondary microcephaly category (NMNH: 242498, 264595, 266109, 266454). It should be noted, however, that several skulls assigned to the secondary microcephaly group overlap in size with the healthy human population samples, in particular those from the Andaman Islands that are noted for their small overall body size.

A few immature specimens were included to maximize sample sizes. One ME hypothyroid individual was a juvenile (un-erupted M3). This specimen increased the median/average Procrustes distance for the hypothyroidism groups but only by a negligible amount (Table 2). Three primary microcephalic individuals may be juveniles (unfused spheno-occipital synchondroses and un-erupted M3s) while one primary and one secondary microcephalic were older subadults (partially fused synchondroses). The juvenile/subadult microcephalics either did not affect values or resulted in slightly *smaller* values for the average/median Procrustes distances in that sample (Table 2). Ultimately, these few juveniles extended the variation in pathological samples, thereby increasing the likelihood that LB1 might plot within these groups. Furthermore, neurocranial growth (as represented by head circumference) ceases around 4 years of age in patients with microcephaly [67], and all specimens were judged to be ≥4 years based on dental eruption.

**Table 2:** Summary statistics for Procrustes distances between LB1 and each group, with individual distances included for each fossil hominin.

LB1 to:	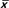	*s*	Range
Laron Syndrome			0.202
ME hypothryoidism^1^	0.157	0.018	0.116–0.182
All hypothyroidism^2^	0.156	0.016	0.116–0.182
Healthy humans	0.147	0.015	0.117–0.190
All microcephaly^3^	0.139	0.016	0.116–0.179
Primary microcephaly^4^	0.136	0.017	0.116–0.179
*Homo habilis* (KNM-ER 1813)			0.123
Mid-Pleistocene *Homo*	0.119	0.008	0.107–0.131
Sima de los Huesos 5			0.107
La Ferrassie			0.115
Dali			0.117
La Chapelle aux Saints			0.122
Omo Kibish II			0.122
Kabwe			0.131
*Homo erectus*	0.108	0.009	0.094–0.121
D 2700			0.094
D 2280			0.096
Ngandong 12			0.098
Zhoukoudian 11			0.101
Sangiran 17			0.104
KNM-ER 3733			0.105
D 3443			0.112
KNM-ER 3883			0.113
Sambungmacan 3			0.113
Sambungmacan 1			0.113
BOU-VP-2/66 (Daka)			0.114
Ngandong 6			0.116
Ngandong 11			0.121

1, Excluding the juvenile specimen: 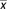 = 0.154, *s* = 0.017.

2, Excluding the juvenile specimen: 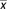 = 0.154, *s* = 0.015.

3, Excluding the juvenile/subadult specimens did not affect 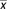 or *s*.

4, Excluding the juvenile/subadult specimens: 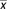 = 0.139, *s* = 0.019.

## Methods

Three-dimensional (3D) landmark data were acquired for the majority of the sample using a Microscribe 3D point digitizer. For some pathological specimens, identical landmark data were collected from surface renderings (based on CT or laser surface scan data) using AMIRA 5.3.2 software (Table 1). Landmarks were chosen to capture the shape of the cranial vault and basicranium (Table 3), and are a subset of a larger landmark protocol described elsewhere [68]. In some cases, missing landmarks were estimated based on reflected relabeling [3], [69], morphology preserved in the immediate vicinity of the missing landmark [see also 70], or by reference to CT scan data [see ref. 3 for additional details about landmark reconstruction in LB1 and other fossil hominins]. As documented elsewhere [1], LB1 suffered damage to its cranium during excavation, including the glabellar and interorbital regions, as well as the left supraorbital region. For this reason, we excluded landmarks from these areas. Kaifu et al. [31] provided a detailed assessment of suture and osteometric landmark positions for LB1 based on CT data. Their basic approach to locating osteometric landmarks in cases where the sutures were difficult to discern (lambda, asterion) or there was damage (bregma) was to trace the visible portions of the sutures (or the raised region indicating where the suture would have been located) to their apparent intersections. We used the same approach, and their descriptions of the landmarks appear to coincide closely with our own assessments.

**Table 3:** Landmarks used in this study.

Landmark	Definition
Inion	Point at which superior nuchal lines merge in midsagittal plane
Lambda	The apex of the occipital bone at its junction with the parietals, in the midline
Bregma	Posterior border of the frontal bone in the midsagittal plane
Dacryon	Point where lacrimo-maxilary suture meets the frontal bone
Supraorbital notch	Point of greatest projection of notch into orbital space, taken on medial side of notch
Frontomalare temporale	Point where the fronto-zygomatic suture crosses the temporal line
Frontomalare orbitale	Point where the fronto-zygomatic suture crosses the inner orbital rim
Mid-torus inferior	Point on inferior margin of supraobrital torus roughly at the middle of the orbit (on superior margin of orbit)
Mid-torus superior	Point on superior aspect of supraorbital torus, directly above mid-torus inferior on anterior aspect of torus
Anterior pterion	Where coronal suture intersects spheno-frontal or spheno-parietal suture
Porion	Uppermost point on the margin of the external auditory meatus
Auriculare	Point vertically above the center of the external auditory meatus at the root of the zygomatic process
Frontotemporale	Point where the temporal line reaches its most antero-medial position on the frontal
Asterion	The common meeting point of the temporal, parietal, and occipital bones, on either side
Opisthion	Midline point at the posterior margin of the foramen magnum
Tympano-mastoid fissure	Point on lateral border of the tympano-mastoid fissure
Medial petrotympanic crest	Most medial point of petrotympanic crest at level of carotid canal
Lateral petrotympanic crest	Lateral origin of petrotympanic crest; if the petrotympanic crest splits, point is taken posteriorly
Stylomastoid foramen	Posterior border of sylomastoid foramen
Postglenoid process	Infralateral-most point posterior to glenoid fossa and anterior to ectotympanic tube (postglenoid tuberosity or crest)
Inferior entoglenoid	Most inferior point on the entoglenoid pyramid
Lateral articular fossa	Deepest point on the lateral margin of the articular eminence (root of the articular eminence)
Temporo-sphenoid suture	Point where temporo-sphenoid suture passes from squama to cranial base (often on infratemporal crest)
Mid-temporal	Point on the temporal squama midway between temporo-sphenoid and parietal notch (calculated from semilandmark data)

All landmark configurations were superimposed using generalized Procrustes analysis [71], and superimposed coordinates were subjected to principal component analysis (PCA) to reduce the dimensionality of data and to visualize the main axes of variation. Ordinations were computed in the SAS 9.2 software package; shape differences associated with each PC axis were visualized using the EVAN Toolkit software version 1.0 (for surfaces) and Morphologika2 v2.5 software package (for wireframes) [72]. Shape differences were exaggerated for easier visualization by plotting shapes at PC scores corresponding to −0.1/+0.1 along each axis; the exception was PC 1 which was visualized at the highest positive score (which was >0.1). Centroid size (the square root of the sum of squared distances of each landmark to the centroid), a proxy of overall size, was used to explore allometric variation along particular PC axes using ordinary least-squares (OLS) regression of PC scores on log(centroid size). Pairwise Procrustes distances, which correspond to shape differences between pairs of specimens in full shape space, were computed between LB1 and all other specimens in the Morphologika2 2.5 software package.

A between-group PCA was also performed to investigate differences in shape among groups, as well as the affinities of LB1 relative to these groups [73]. The PC axes were calculated from the covariance matrix computed from means of the following groups: healthy humans, humans with primary microcephaly, humans with ME hypothyroidism, *H. erectus*, and mid-Pleistocene *Homo* (pooled *H. heidelbergensis* and *H. neanderthalensis* samples) (Table 1). All specimens comprising these groups, as well as LB1, were then projected onto these axes. Therefore, the principal component axes in this analysis reflect shape differences among group means rather than among all individuals in the sample. Creating a single mean shape for the primary microcephaly and ME hypothyroidism groups may be problematic given their documented heterogeneity. However, the standard PCA demonstrates that neither of these groups is randomly distributed through morphospace. Rather, they each occupy distinct morphospace regions, indicating shared components of cranial shape among individuals in each pathological group, despite some degree of heterogeneity.

## Results

**Figure 1:**
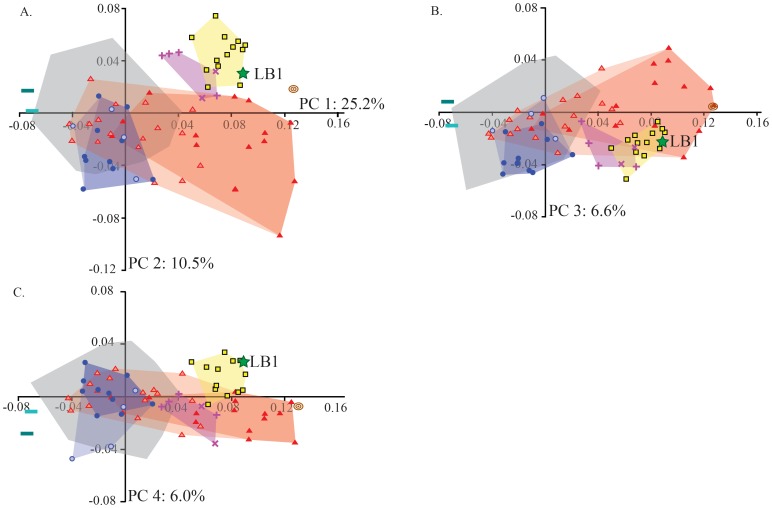
Principal component analysis of neurocranial shape with minimum convex polygons drawn as shaded regions around each group. (A) The shape of the LB1 neurocranium is distinct from that of healthy humans and humans with hypothyroidism or Laron syndrome on PC 1, and within the *Homo erectus* distribution on PC 2. (B) LB1 overlaps both fossil *Homo* and microcephalic humans on PCs 1 and 3, but (C) again groups with *H. erectus* on the fourth component. Figure legend: LB1: green star; *H. habilis*: brown target; *H. erectus*: yellow squares; Mid-Pleistocene *Homo*: purple crosses; Neanderthals: purple Xs; Primary microcephaly: red triangles; Secondary microcephaly: pink triangles; ME hypothyroidism: blue circles; Sporadic hypothyroidism: light blue circles; Laron syndrome: dark aqua dash; “Pituitary dwarf”: light aqua dash. For clarity, only the gray convex polygon is shown for the healthy human sample rather than individual data points. Light blue and light pink polygons extend the hypothyroid and microcephaly distributions to include the sporadic hypothyroid and secondary microcephaly specimens, respectively.

**Figure 2:**
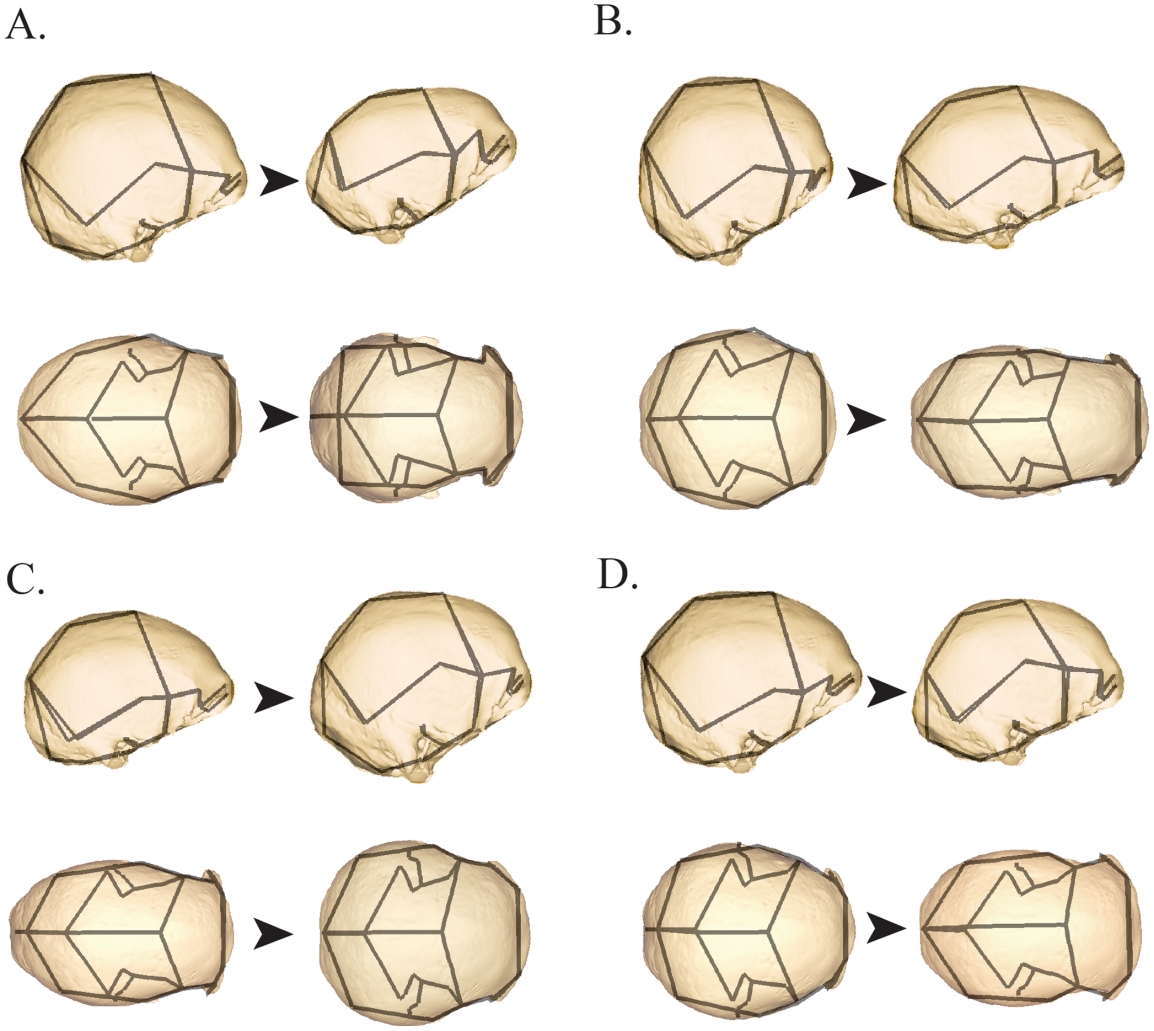
Shape differences associated with the first four components of the PCA based on the full sample and illustrated in Figure 1. Wireframes are superimposed on warped surfaces to illustrate shape differences from the negative (left) to positive (right) ends of (A) PC 1, (B) PC 2, (C) PC 3 and (D) PC 4.

Examination of the first four PCs based on the full sample, which together account for almost half of the total shape variance, yields three important observations relevant to LB1’s status. Firstly, the shape of the LB1 neurocranium is very different from those of humans with Laron syndrome and ME hypothyroidism (PC 1 in Fig. 1A). Secondly, there are convergences in cranial shape between archaic *Homo* species and modern humans with microcephaly (particularly primary microcephaly) (PCs 1 and 3 in Fig. 1B) related to the latter group’s low cranial vault height, angled occipital, low temporal squama, and increased postorbital constriction (Fig. 2A,C). Thirdly, despite these convergences, archaic *Homo* and LB1 are differentiated from the primary microcephaly sample by virtue of their longer and relatively lower cranial vault and more robust supraorbital torus (PC 2 in Fig. 1A, Fig. 2B). There is also minimal overlap between *H. erectus* and microcephalic modern humans on PC 4, with LB1 again diverging from the latter in the direction of *H. erectus* due to a combination of lower frontal profiles and more angled occipitals (Fig. 1C, Fig. 2D). Specimen distributions on PCs 1, 2 and 4 do not reflect substantial allometric variation, as indicated by OLS regression of PC scores on the natural logarithm of centroid size (PC 1: *R^2^* = 0.08, *p*<0.001; PC 2: *R^2^* = 0.13, *p*<0.001; PC 4: *R^2^* = 0.08, *p*<0.001); there is no significant size-shape relationship on PC 3 (*p* = 0.401). This is not surprising, perhaps, given that the centroid size of the LB1 neurocranium is within the range of the microcephalic individuals and smaller than all of the *H. erectus* and *H. habilis* specimens, yet it nevertheless scores within the fossil hominin range on both PCs 2 and 4.

**Figure 3:**
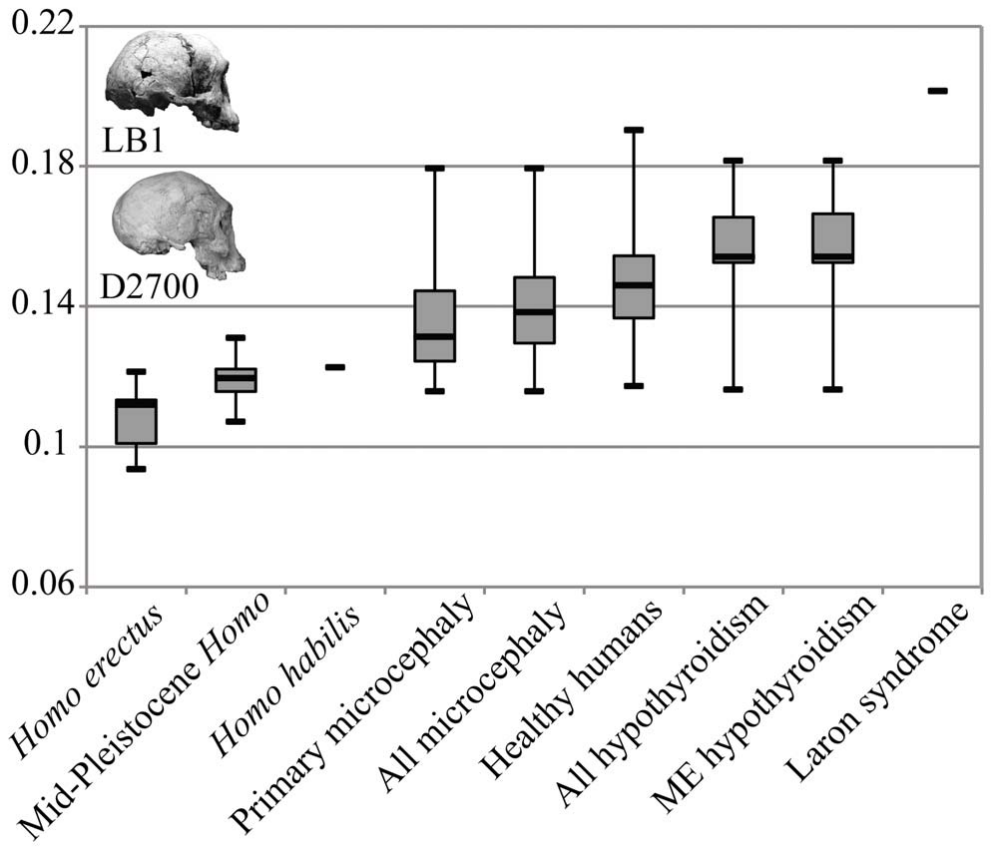
Box-and-whisker plot of Procrustes distances between LB1 and each of the other specimens. **Procrustes distances are calculated based on the entire set of neurocranial landmarks.** In median distance, LB1 is most similar to the *H. erectus* sample and most dissimilar to the Laron syndrome individual. LB1 has the shortest distance to the D2700 *H. erectus* fossil from Dmanisi, Georgia. Boxes are bounded by 25th and 75th percentiles, with medians indicated by the solid lines; whiskers denote minimum and maximum distances in the sample to LB1. LB1 is closest in shape space to a Georgian *H. erectus* specimen, D2700, pictured below LB1 in the inset photographs (not to scale).

Procrustes distances computed in the full shape space corroborate results illustrated in the lower dimensional PC space: LB1 is most similar to *H. erectus* and other archaic *Homo* in its overall neurocranial shape (Fig. 3). The Dmanisi specimens D2700 and D2280 are most similar in shape to LB1 and substantially closer than any of the modern human normocephalic or pathological specimens (Table 2). Among all of the modern human samples, both healthy and pathological, those specimens closest to LB1 were only as similar as the most distant specimens of *H. erectus* (Table 2).

**Figure 4:**
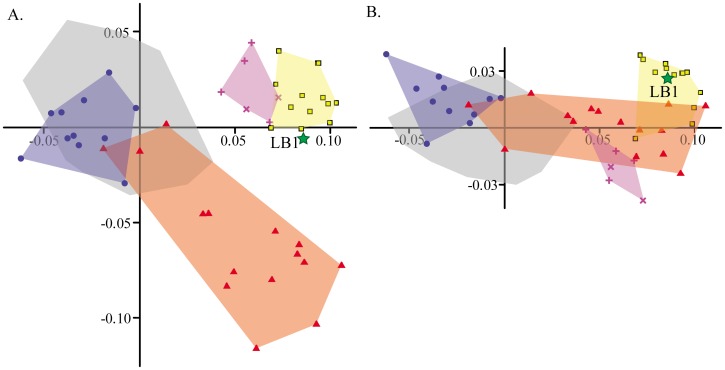
Between-group PCA of neurocranial shape. Analysis was based on five *a priori* groups: *H. erectus sensu lato*, middle-Pleistocene *Homo*, healthy modern humans, humans with ME hypothyroidism and humans with primary microcephaly. When individual specimens are projected onto axes computed from group means, LB1 plots closest to the group centroid for *H. erectus* on (A) PCs 1 and 2 and (B) PCs 1 and 4. See Figure 1 for Legend.

Results of the between-groups PCA, which better depicts group separation than a standard PCA [73], offers clear confirmation that LB1’s neurocranial affinities lie with *H. erectus* (Fig. 4). Healthy modern humans as well as those with ME hypothyroidism (and some with primary microcephaly) are quite distinct from the remaining samples on PC 1, as in the standard PCA. The biggest difference between this ordination compared to the standard PCA is the clearer separation of the fossil *Homo* sample from the remainder of the primary microcephaly group on the second component and the strong association of LB1 with the fossil *Homo* group. The third component (not figured) adds little information beyond partial separation between healthy humans and those with ME hypothyroidism. The majority of the *H. erectus* sample is further distinguished from the *H. heidelbergensis*/*H. neanderthalensis* group on PC 4, and LB1 plots closest to the *H. erectus* centroid.

## Discussion

This study demonstrates that the shape of the LB1 neurocranium is outside the ranges of variation documented here for the ME hypothyroidism specimens and distinct from the specimen with Laron syndrome – strongly suggesting that neither condition is the underlying factor explaining LB1's neurocranial shape [see also 43,45]. These results are consistent with descriptions of the cranial phenotypes associated with cretinism [58] and Laron syndrome [44], both of which differ markedly from the morphology exhibited by LB1. With regard to ME hypothyroidism, however, much of the evidence favoring this diagnosis derives from postcranial rather than cranial anatomy. Such arguments are beyond the scope of this paper, and are reviewed completely elsewhere [18,19,34, but see 27,43]. It is worth noting that our hypothyroidism sample is relatively variable despite its common geographic (and possibly genetic) origin in the Swiss Alps. Individuals with endemic hypothyroidism are often categorized as neurological or ME “cretins” based on whether their symptoms are primarily neurological or both neurological and somatic (including short stature), respectively. However, this apparent dichotomy may actually represent the extremes of a continuum based on timing and severity of iodine deficiency pre- and post-natally [42], and the variability of our hypothyroidism sample may reflect the presence of individuals at different points along this continuum. Importantly, none of these individuals, including those of small body size, resembles LB1. Hence, our results present a new line of evidence in this debate: the magnitude of differences in cranial anatomy between LB1 and the healthy human sample far exceeds the differences between the cretin and healthy human samples. This suggests that ME hypothyroidism, at least in a population of modern humans, is unlikely to result in the distinctive cranial anatomy found in LB1.

The between-group PCA, which emphasizes differences between group means rather than inter-individual variability, showed a clear affiliation between LB1 and extinct *Homo* species, particularly *H. erectus*. The distinct distribution of microcephalics in this analysis suggests that there are common components of shape shared by nearly all specimens in this group (Fig. 4), likely related to the premature termination of brain (and therefore neurocranial) growth. Where fossil hominins and the remaining microcephalics differ from this common pattern, they do so in very different ways (the latter more resembling modern humans). Therefore, our results confirm earlier studies of both ectocranial and endocranial morphology of LB1, which found it to be distinct from modern human microcephalic conditions on the basis of much smaller pathological samples [4], [5], [47].

One confounding factor in determining the affinities of LB1 is that some features that are characteristic of fossil hominins are also found in modern pathological specimens (e.g., Fig. 1B): low midline cranial profile, angled occipital bone, low temporal squama, and increased postorbital constriction. The presence of these same characteristics in the LB1 skull is therefore of limited utility for establishing its affinities with *either* group. Yet despite superficial resemblances among these samples, there are clear metric differences between fossil *Homo* and the microcephalic crania, and in these features LB1 consistently aligns with the former. An elongated and low cranial vault, relatively small parietal bones, and expanded supraorbital torus add to the list of LB1 features that are typical of many fossil *Homo* species, but not characteristic of any of the humans exhibiting the pathologies examined here.

In the present study, LB1 shows particular affinities in neurocranial shape with the Dmanisi hominins, including the subadult cranium D2700 and adult neurocranium D2280 [see also 29]. These relationships hold regardless of whether modern humans are included in the analysis (for example, if one considers Procrustes distances). The Dmanisi fossils date to the beginning of the *H. erectus s.l.* time range (∼1.7–1.8 Ma), and their cranial morphology is similar to other early *H. erectus* from Africa [68], [74]. Although D2700 is a subadult (as judged by its unfused spheno-occipital synchondrosis and M3s that are erupted but not in occlusion [74], [75]), its overall cranial shape is within the *H. erectus* range [3] and its neurocranium conforms to expectations for an adult *H. erectus* of the same size [76]. Nevertheless, interpreting this affinity is complicated by the fact that overall neurocranial shape is correlated with cranial size within the *H. erectus* lineage [3], [76], [77]. Therefore, the similarities between LB1 and Georgian *H. erectus* could be due to, or enhanced by, their small size rather than a close phylogenetic relationship. The presence of static allometry in *H. erectus* also means that we cannot exclude the possibility that *H. floresiensis* represents a dwarfed lineage of Asian *H. erectus* on the basis of neurocranial shape. More recent support for this hypothesis was offered by Kaifu et al. [31] in the form of detailed metric and nonmetric resemblances between the LB1 cranium and early Indonesian *H. erectus* from Sangiran and Trinil. Moreover, a recent analysis suggested that as little as 10–30% of brain size reduction in LB1 remains to be explained above and beyond body size reduction from a female early Indonesian *H. erectus* based on intraspecific scaling of brain mass-to-body mass in a broad geographic sample of modern humans [15].

Our study was not able to fully evaluate the likelihood of a pre-*H. erectus* ancestry for *H. floresiensis* since only a single *H. habilis* specimen was complete and undamaged enough to be included in our analyses. Nevertheless, the differences observed here between LB1 and KNM-ER 1813 (*H. habilis*) correspond to more derived features in LB1 that distinguish it from a larger *H. habilis* sample analyzed by Kaifu and colleagues [31] (e.g., LB1 has a wider cranial vault and more flexed occipital bone). This may suggest that the cranial morphology of LB1 is more derived than *H. habilis* in the direction of *H. erectus s.l.*

### Conclusions

Our analyses corroborate the previously suggested link between LB1 and fossil *Homo* and support the attribution of this specimen to a distinct taxon, *H. floresiensis*. Furthermore, the neurocranial shape of *H. floresiensis* closely resembles that of *H. erectus s.l.* and particularly specimens of early Eurasian *H. erectus*, although it is unclear whether this latter affinity is best attributed to a close phylogenetic relationship or to a size-related convergence in shape. These results also counter the hypotheses of pathological conditions [2], [43], [45] as the underlying cause of the LB1 neurocranial phenotype, with the possible exception of posterior deformational plagiocephaly, a condition without significant adverse health effects [8].
